# Kinetic stability analysis of protein assembly on the center manifold around the critical point

**DOI:** 10.1186/s12918-017-0391-7

**Published:** 2017-02-02

**Authors:** Tatsuaki Tsuruyama

**Affiliations:** Department of Pathology, Kyoto University, Graduate School of Medicine, Yoshida-Konoe-Cho 1, Kyoto, Kyoto Prefecture 606-8501 Japan

**Keywords:** Protein assembly, Nonlinear kinetics, Fluctuations

## Abstract

**Background:**

Non-linear kinetic analysis is a useful method for illustration of the dynamic behavior of cellular biological systems. To date, center manifold theory (CMT) has not been sufficiently applied for stability analysis of biological systems. The aim of this study is to demonstrate the application of CMT to kinetic analysis of protein assembly and disassembly, and to propose a novel framework for nonlinear multi-parametric analysis. We propose a protein assembly model with nonlinear kinetics provided by the fluctuation in monomer concentrations during their diffusion.

**Results:**

When the diffusion process of a monomer is self-limited to give kinetics non-linearity, numerical simulations suggest the probability that the assembly and disassembly oscillate near the critical point. We applied CMT to kinetic analysis of the center manifold around the critical point in detail, and successfully demonstrated bifurcation around the critical point, which explained the observed oscillation.

**Conclusions:**

The stability kinetics of the present model based on CMT illustrates a unique feature of protein assembly, namely non-linear behavior. Our findings are expected to provide methodology for analysis of biological systems.

## Background

Numerical simulation based upon multi-parametric kinetic equations is the principal methodology for the analysis of the behavior of biological systems. Researchers often encounter a number of parameters in the governing equations of the system. Here, we introduce the center manifold theory (CMT) for simplification of the study of dynamic biological systems. CMT provides mathematical prescription for carrying out reduction of the number of parameters near the steady state, as well as information regarding the stability of the steady state. As a result, simulation is oriented to illustrate behavior around the critical point, at which system behavior drastically changes in the qualitative structure. The observable change is termed bifurcation, and the threshold values of the parameters are referred to as critical values or bifurcation values. The aim of this study was to provide a simple algorithm for the application of CMT to multi-parametric kinetic equations, in order to clearly illustrate the behavior of the biological system. The CMT has been applied to the Lotka-Volterra model of predator–prey system to provide important simulation results [1, 2]. In addition, several pioneering studies have applied CMT to neural network analysis [3]. Time-delay and diffusive effects play important roles in bifurcation phenomena [1, 4]. However, to date, there are few applications of the CMT to biochemical reaction models. We previously reported a model of cell signaling systems using non-linear kinetics and demonstrated the phase transition phenomenon via a numerical simulation [5].

Pivotal protein-protein interactions during cytoskeleton formation were selected as the application model for the present CMT method. Among the interactions between protein monomers, tubulin and actin polymerization are well-known events that have been analyzed using the numerical method [6–10]. The physical robustness of the cytoskeleton is based on the biophysical properties of actin and tubulin. In particular, various mathematical models have been proposed to explain the kinetic behavior of tubulin assembly [6–11]. A theory of polymerization of macromolecules has been established on the basis of the kinetic model of aggregation [12, 13]. Oosawa and Asakura previously reported that polymerization is similar to micelle formation or crystallization, and that there is a critical monomer concentration above which monomers effectively polymerize. The authors additionally suggested that the nucleation step represents the rate-limiting step for polymerization. Nucleation and growth occur in parallel during the progression of polymerization. There is a gap in free energy change between initial nucleation and progression of linear polymerization [13]. The stable nucleus for polymerization consists of trimers or tetramers, and the growth of aggregates through elongation/ dissociation follows the formation of a thermodynamically unfavorable size of the nucleus. In the current study, we focused on the polymerization in the absence of *de novo* nucleation and the interaction between polymer and monomer (PM) interaction.

For stable growth, the lifespan of tubules is controlled by a guanidine triphosphate (GTP)-cap that forms at their ends [14]. The structure and motility of growing tubules is influenced by intrapolymeric Brownian motion and fluctuation; this provides elasticity to the microtubules [15]. Polymerization/de-polymerization is controlled by binding of adenosine triphosphate (ATP)/GTP, resulting in the assembly of monomeric proteins. The intermittent transition between slow growth and rapid shrinkage in polymeric assemblies of microtubules is termed *dynamic instability* [14]. Numerous models have been proposed to explain this instability; in particular, Zapperi and Mahadevan successfully identified two parameters: a structural mechanical parameter that characterizes the ratio of longitudinal to lateral interactions in an assembly, and a kinetic parameter that characterizes the ratio of timescales for growth and conformation change. These parameters serve to demarcate a region of uninterrupted growth from that of collapse [16].

In the current study, we consider a model assembly system that shows the unstable dynamics of assembly around the critical concentration of ATP/GTP. The present model utilizes CMT for describing the behavior of monomers in the solvent and polymer for simplification of analysis. We applied a kinetic model that unifies *de novo* nucleation and growth by considering the monomer-monomer interactions as a diffusion process. In addition, the diffusion process of the monomeric protein has been considered from the perspective of non-linearity. According to Fick’s law, the continuity of monomer concentration of *c*_*i*_ (*i* = 1*, n*) including chemical reaction items, may be described using diffusion coefficients *D*_*i*_, kinetic coefficients *k*_*i*_, and the concentrations of individual compounds *c*_*i*_. Protein assembly is limited by the slow diffusion rate of monomer proteins, which is a diffusion-rate limiting aggregation process. Therefore, diffusion items and reaction items cannot necessarily be separated; therefore, we described kinetic rate of *c*_*i*_ as follows [8]:

1 $$ \frac{d{c}_i}{dt}={k}_i{D}_i{c}_i+f\left({c}_i\right) $$

Here, the first item on the right represents the diffusion rate. The second item, *f* (*c*_*i*_), denotes the function of kinetic rate of reactions other than the diffusion process. *k*_*i*_ represents a coefficient.

## Methods

Numerical simulation Numerical calculations were performed using Mathematica 8 (Wolfram Research, Inc., Champaign, IL).

## Results

### General formulation of an assembly

The model consists of several steps: (i) the monomer achieves an interactive state by binding a cofactor (ATP/GTP) that provides the monomer with the ability to interact; (ii) the monomer itself possesses the ability to hydrolyze the cofactor and lose assembly activity; (iii) the monomer has the ability to exchange the inactive hydrolyzed cofactor (ADP/GDP) with an active non-hydrolyzed one; and (iv) ATP/GTP are supplied continuously from the external environment. The second requirement indicates a self-limiting property of the monomer that causes dynamic instability during monomer-monomer interaction. When examining protein interaction kinetics, analysis of the fluctuation in monomer concentrations was performed using Mathematica 9.

### Protein interaction kinetics

**Fig. 1:**
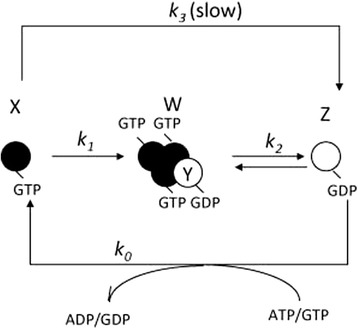
Scheme of monomer interaction. Individual globules or oblongs represent monomers *X, Y, Z,* and oligomer *W*. Kinetic coefficients, *k*_*0*_, *k*_*1*_ *, k*_*2*_ *,* and *k*_*3*_ are shown next to the arrows. Outside and inside signify the outside and inside of the cell, respectively. *Y* is located at the end of the oligomer *W*

The model scheme is shown in Fig. 1. There are three types of monomer: ATP/GTP-binding monomer X, ADP/GDP-binding monomer Y in the oligomer (W), and the released ADP/GDP-binding monomer Z. X has the higher assembly activity, and Y and Z have lower assembly activity. We set the oligomer concentration W to be a constant, as *de novo* assembly is considered much slower than monomer interaction in the steady state [11–14]. The individual steps are shown below:

First, X associates with the assembly nucleus W to be Y at the end of W.

2 $$ X+W\to W+Y\kern0.5em \left({m}_1; kinetic\; coefficients\right) $$

In the next step, the intermediate species *Y* is released to be *Z*:

3 $$ Y\to Z\kern0.5em \left({m}_2\right) $$

*Z* recovers its interaction activity by exchanging the active cofactor ATP/GTP (*P*) for the inactive cofactor ADP/GDP (*P*’), returning to *X* (see Fig. 1):

4 $$ Z+P\to X+{P}^{\prime}\kern0.5em \left({k}_0\right) $$

In addition, direct slow conversion is supposed:

5 $$ X\ \to\ Z\kern0.5em \left({k}_3\right) $$

The kinetic equations were set according to the simple reaction cascade described above. We obtained equations for the protein interaction kinetics using the diffusion coefficient:

6 $$ dX/dt=-{m}_1WX+{k}_0PZ-{k}_3X $$

7 $$ dY/dt={m}_1WX-{m}_2Y $$

8 $$ dZ/dt={m}_2Y-{k}_0PZ+{k}_3X $$

In addition, the total concentration of the monomer is maintained constant.

9 $$ X+Y+Z=M $$

M*,* which represents the total concentration of the monomeric proteins, is maintained constant. A simple consideration of the diffusion-limited step implies that, when the kinetic rate can be described according to Fick’s law using the diffusion coefficients *D*_*X*_, *D*_*Y*_ and *D*_*W*_ then [17–19]:

10 $$ {m}_1\propto \left({D}_X+{D}_W\right)/2\simeq {D}_X/2 $$

11 $$ {m}_2\propto \left({D}_W+{D}_Y\right)/2\simeq {D}_Y/2 $$

As the oligomer diffusion rate is small, we set *D*_*X*_, *D*_*Y*_ > >*D*_*W*_. Therefore, *m*_*1*_, and *m*_*2*_ are substantially proportional to *D*_*X*_ and *D*_*Y*_, respectively. Accordingly. kinetic coefficients *k*_*1*_ and *k*_*2*_ were defined as the proportional coefficients below:

$$ \begin{array}{l}{m}_1\triangleq {k}_1{D}_X\\ {}{m}_2\triangleq {k}_2{D}_Y\end{array} $$

Rewriting (6), (7), and (8) using (10) and (11),

12 $$ dX/dt=-{k}_1{D}_XWX+{k}_0PZ-{k}_3X $$

13 $$ dY/dt={k}_1{D}_XWX-{k}_2{D}_YY $$

14 $$ dZ/dt={k}_2{D}_YY-{k}_0PZ+{k}_3X $$

In the above equations, *k*_*1*_ and *k*_*2*_ represent the kinetic coefficients for the addition of the monomer to the oligomer and the release of the monomer from the oligomer, respectively.

In order to obtain the monomer concentration at the steady state of the reaction system, the right-hand side of Eqs. (12), (13), and (14) were set to be equal to zero and Eq. (9) were used to give:

15 $$ {X}_e=\frac{D_Y{k}_2Mp}{D_X{k}_3{k}_2+{D}_Y{k}_2p+{D}_X{D}_Y{k}_1{k}_2W+{D}_X{k}_1 pW}\sim \frac{D_Y{k}_2M}{D_Y{k}_2+{D}_X{k}_1W} $$

16 $$ {Y}_e=\frac{D_X{k}_1MpW}{D_Y{k}_3{k}_2+{D}_Y{k}_2p+{D}_X{D}_Y{k}_1{k}_2W+{D}_X{k}_1 pW}\sim \frac{D_X{k}_1MW}{D_Y{k}_2+{D}_X{k}_1W} $$

17 $$ {Z}_e=\frac{D_Y{k}_2M\left({k}_3+{D}_X{k}_1W\right)}{D_Y{k}_3{k}_2+{D}_Y{k}_2p+{D}_X{D}_Y{k}_1{k}_2W+{D}_X{k}_1 pW}\sim \frac{D_Y{k}_2M\left({D}_X{k}_1W\right)}{D_Y{k}_2p+{D}_X{k}_1 pW}\sim 0 $$

In the above approximation, we omitted *D*_*X*_ *D*_*Y*_ and *k*_*3*_ as the diffusion coefficients and the direct conversion rate of *X* into *Z* is small.

### Fluctuation of diffusion coefficient

Next, we considered the fluctuations of participant proteins using small letters *x, y*, and *z*:

18 $$ X={X}_e+x,Y={Y}_e+y,Z={Z}_e+z $$

In Eq. (14), the subscript ‘e’ signifies values at the steady state.

In an assembly, monomers associate with other monomers. From Eq. (9),

19 $$ x+y+z=0 $$

Therefore, the fluctuation *y* may be represented using −*x−z.* The fluctuation kinetics are thus provided by two parameters, namely *x* and *z*.

Given the nonlinearity during diffusion, we assume kinetic instability in the monomer-monomer interaction, and that the sensitivity of the assembly in response to environmental change may be evaluated. Indeed, the diffusion coefficient *D*_*i*_ of one macromolecule in the solution may generally be represented using the fluctuation concentration *c*_*i*_:

20 $$ {D}_i={D_i}^0-\varSigma {\alpha}_{ij}{c}_i={D_i}^0-d{D}_i\kern0.5em \mathrm{with}\kern0.28em d{D}_i\equiv \varSigma {\alpha}_{ij}{c}_i\left(1\le i\le 3,i=X,Y,Z\right) $$

*c*_*j*_ denotes the concentration of the solute, *α*_*i*_ is a coefficient, and *D*_*i*_^*0*^ is the diffusion coefficient when the fluctuation of monomeric protein is negligible. The dependence of the diffusion coefficient on the protein concentration has been reported [20, 21]. O’Learly reported that diffusion coefficients of proteins linearly decrease in proportion to the concentration, when the latter is sufficiently small. The fluctuation of the diffusion coefficient is obtained by considering the dependence of the coefficients on the concentration of the monomer from Eq. (20) [8]:

21 $$ d{D}_X=\alpha x-\beta z $$

22 $$ d{D}_Z=\gamma x-\delta z $$

Here, the fluctuation term *αx* (*α* >0) and *γx* (*γ* >0) contributes to a decrease in *D*_*X*_ and *D*_*Z*_, as higher assembly activity reduces diffusion. In contrast, an increase in the fluctuation terms *βz* (*β* >0) and *δz* (*δ* >0 serves to increase the diffusion coefficients *D*_*X*_ and *D*_*Z*_, as lower interaction or assembly activity increases diffusion. When the assembly activity of *Z* is lower, the fluctuation item *δz* is negligible, in accordance with the fluctuation kinetic equations given by (19), Eqs. (12), (14), (21), and (22):

23 $$ dx/dt=-{k}_1W\left({D}_X-\alpha x+\beta z\right)\left({X}_{\mathrm{e}}+x\right)+{k}_0P\left({Z}_e+z\right)-{k}_3\left({X}_{\mathrm{e}}+x\right) $$

24 $$ dz/dt={k}_3\left({X}_{\mathrm{e}}+x\right)-{k}_2\left({D}_Y-\gamma x+\delta z\right)\left({Y}_{\mathrm{e}}+y\right)-{k}_0P\left({Z}_e+z\right) $$

Here, *y*, fluctuation of intermediate species *Y* is negligible as the value is sufficiently small. In addition, we used the following equations to describe the balance in detail:

25 $$ -{k}_1{D}_XWX+{k}_0PZ-{k}_3X=0 $$

and

26 $$ {k}_2{D}_YY-{k}_0PZ+{k}_3X=0 $$

To simplify the notation in (23) and (24), we set:

27 $$ \begin{array}{l}{k}_1{D}_XW={D}_1,{k}_1{D}_XW\;\alpha =a,\kern0.5em {k}_1{D}_XW\beta =b\\ {}{k}_2\gamma =c,\kern0.5em {k}_0P=p,\;{k}_3 = k\end{array} $$

and obtained:

28 $$ dx/dt=-\left({D}_1-a{X}_{\mathrm{e}}-k\right)x+\left(-b{X}_{\mathrm{e}}+p\right)z+a{x}^2-bxz $$

29 $$ dz/dt=\left(k-c{Y}_{\mathrm{e}}\right)x-pz+c{x}^2+cxz $$

Eqs. (28) and (29) represent a master equation for the application of CMT.

### Calculus simulation of concentration oscillations

For analysis of the behavior of the system, including multi-parameters, the examination of the linearization of behavior of the system near a steady state provides insights into the qualitative behavior of the system in the neighborhood of the point. In particular, the eigenvalues of the linear part of the governing kinetic equations enable determination of the stability of the system behavior. CMT is a rigorous formulation of this observation that enables the reduction of a large number of parameters [22].

Around the steady state (*x*, *z*) = (0, 0) of Eqs. (28) and (29), the Jacobian matrix of (*dx/dt*, *dz/dt*) is given by:

30 $$ L=\left[\kern1em \begin{array}{cc}-{D}_1+a{X}_e-k\kern1em & \kern1em -b{X}_e+p\kern1em \\ {}\kern1em k-c{Y}_e\kern1em & \kern1em -p\end{array}\kern1em \right] $$

**Fig. 2:**
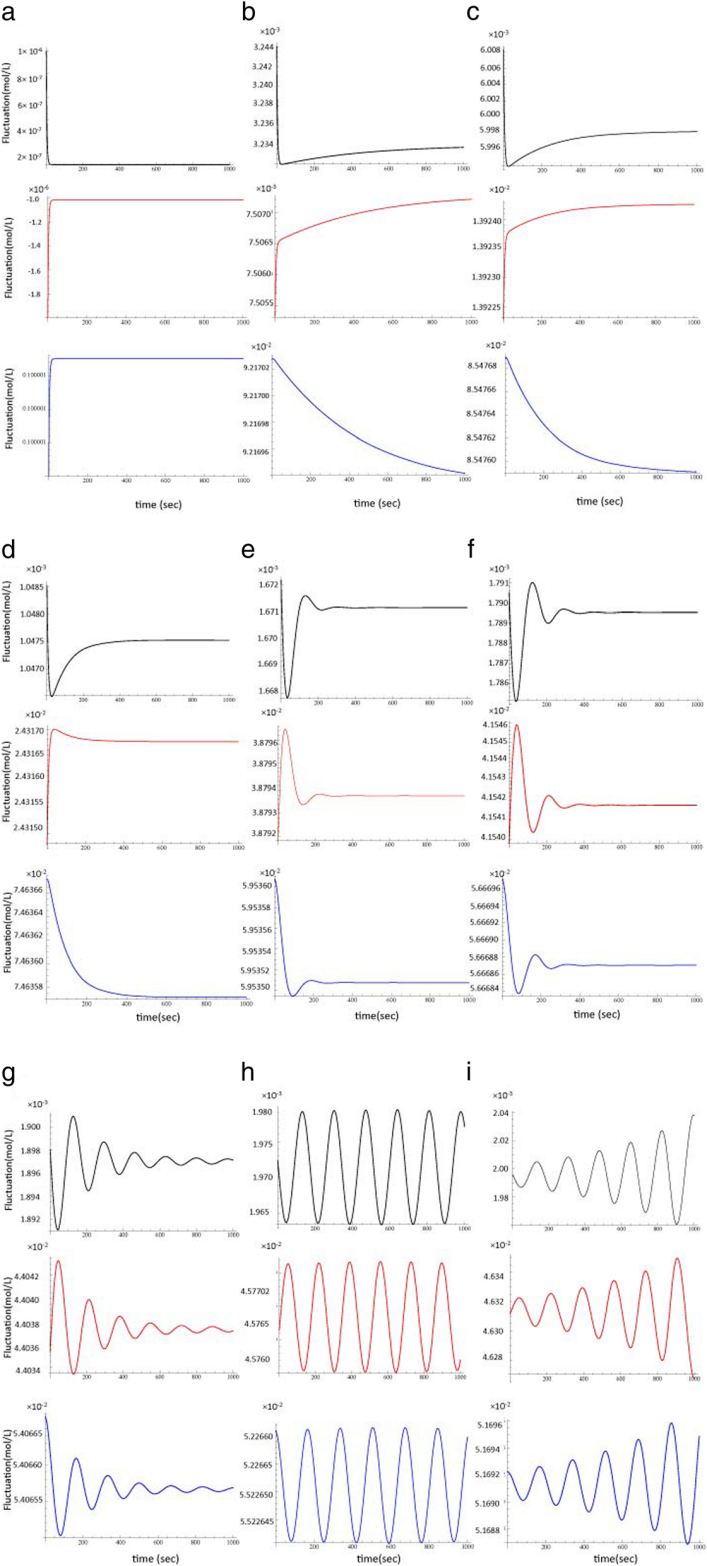
Time-course of the fluctuation in monomer concentrations displays a oscillation. Diffusion of active cofactor binding monomer (*X*) and of inactive cofactor binding monomer (*Z*). *p* is (**a**) 0.000, (**b**) 0.001, (**c**) 0.002, (**d**) 0.004, (**e**) 0.008, (**f**) 0.009, (**g**) 0.01000, (**h**) 0.010705, (**i**) 0.011000. The graphs show plots of *X* (*black*), *Y*(*red*), and *Z* (*blue*). Lines represent the concentration of *X* and *Z*. The horizontal axis represents time (0 ≤ *t ≤* 1000) and the vertical axis represents the concentration of *X* and *Z*. When *p* exceeds 0.01, oscillations are observed. The Mathematica (version 9, Wolfram Research, Inc., Champaign, IL) code for *p* = 0.01 is as follows: p = 0.01 X = ((D2 M p)/(D2 k + D2 p + D1 D2 W + D1 p W)) Y = ((D1 M p W)/(D2 k + D2 p + D1 D2 W + D1 p W)) Z = ((D2 M (k + D1 W))/(D2 k + D2 p + D1 D2 W + D1 p W)) M = 0.1 W = 1 D1 = 0.28 D2 = 0.012061855670103093` a = 150 b = 156 k = 0.005 c = 0.1 d = 0 NDSolve[{Derivative[1][x][t] == − (D1 - a X) x[t] + a x[t]^2 + (p - b X) z[t] - b x[t] z[t] - k x[t], Derivative[1][z][t] == k x[t] + c x[t]^2 + d x[t] z[t] - p z[t], x[0] == 1.`*^-6, z[0] == 1.`*^-6}, {x, z}, {t, 0, 3300}, MaxSteps - >50000] g001 = Plot[{X + x[t]} /. %, {t, 0, 1000}, PlotRange - > All, PlotStyle - > {RGBColor[0, 0, 0]}] g002 = Plot[{Y - x[t] - z[t]} /. %%, {t, 0, 1000}, PlotRange - > All, PlotStyle - > {RGBColor[1, 0, 0]}] g003 = Plot[{Z + z[t]} /. %%%, {t, 0, 1000}, PlotRange - > All, PlotStyle - > {RGBColor[0, 0, 1]}, PlotRange - > All] Show[g001, g002, g003]

Subsequently, the time-course of the monomer concentrations was simulated by substituting appropriate numerical values into Eqs. (28) and (29). The simulation results under the above conditions are shown in Fig. 2. A numerical calculation was performed over a sufficiently long period to evaluate the assembly trend. The steady-state concentrations of *X* and *Z* are given by Eqs. (15) and (17). The critical value of *p*_*c*_ is given by

31 $$ det\left[\mathbf{L}\right]=\left(-{D}_1+a\kern0.28em {X}_e-k\right)\left(-p\right)-\left(k-c{Y}_{\mathrm{e}}\right)\left(-b{X}_{\mathrm{e}}+p\right)=0 $$

Here, the small affix c indicates the *critical point* of ATP/GTP concentration. Next, we conducted a simulation with values of *M* = 0.1, *X*_*e*_ = 0.002, *D*_*1*_ = 0.28, *D*_*2*_ = 0.012, *a* = 150, *b* = 150, *k* = 0.005, *c* = 0.0, and *d* = 0. Solving the above with respect to *p* with substitution of these values in Eq. (31), we find the critical value:

32 $$ {p}_c=0.011 $$

As a result, the fluctuations oscillate between decrease and increase in monomer concentrations, as shown in Fig. 2. When *p* <*p*_*c*_, the fluctuation was found tobe attenuated (Fig. 2d) and the monomer concentration reached a plateau. However, when *p* >*p*_*c*_, the fluctuation was found to diverge (Fig. 2f).

### Evaluation of model stability using the center manifold around the equilibrium state

In order to demonstrate the Hopf-bifurcation around the critical state, in which *p* = *p*_*c*_, we firstly defined the Jacobian matrix ***L***_***c***_ according to (30) :

33 $$ {L}_c=\left[\kern1em \begin{array}{cc}-{D}_1+a{X}_e-k\kern1em & \kern1em -b{X}_e+{p}_c\kern1em \\ {}\kern1em k-c{Y}_e\kern1em & \kern1em -{p}_c\end{array}\kern1em \right] $$

Using the eigenvectors of ***L***_*c*_, [**l**_**1**_ **l**_**2**_], we performed the following coordinate transformation using novel parameters defined by following formulae:

34 $$ \left[\begin{array}{c}\hfill u\hfill \\ {}\hfill v\hfill \end{array}\right]={\left[\begin{array}{cc}\hfill {\boldsymbol{l}}_{\mathbf{1}}\hfill & \hfill {\boldsymbol{l}}_{\mathbf{2}}\hfill \end{array}\right]}^{-1}\left[\begin{array}{c}\hfill x\hfill \\ {}\hfill z\hfill \end{array}\right] $$

With reference to the numerical simulation (Fig. 2), when *D*_*1*_ *, k, Y*_*e*_ *,* and *p*_*c*_ are sufficiently small,

35 $$ \left[\begin{array}{cc}\hfill {\boldsymbol{l}}_{\mathbf{1}}\hfill & \hfill {\boldsymbol{l}}_{\mathbf{2}}\hfill \end{array}\right]=\left[\begin{array}{cc}\hfill -a\ {X}_e\hfill & \hfill 1\hfill \\ {}\hfill\ a{X}_e\hfill & \hfill 1\hfill \end{array}\right] $$

Eigenvalues *λ* of ***L***_*c*_ are

36 $$ \lambda \sim a{X}_e,0 $$

Using (34), we obtained:

37 $$ du/dt={f}_u\left(u,\kern0.28em v\right)=\left({D_1}^2{p}_c+{k}^2v\left(b\left(u-v\right)+av\right)+{D}_1k\left({p}_c\left(u-v\right)+buv\right)\right)/k\left({D}_1+k\right) $$

38 $$ dv/dt={f}_v\left(u,\;v\right)=\Big(-{k}^2v+{D}_1u\left(-{p}_c+bv\right)+k\left({p}_c\left(-u+v\right)+v\left(-{D}_1+bu+\left(a-b\right)v\right)\right)/\left({D}_1+k\right) $$

39 $$ d\varepsilon /dt=0 $$

The center manifold around the critical point (*p* = *p*_c_) is then given as follows.

40 $$ u=h\left(\varepsilon, v\right)={a}_1{v}^2+{a}_2v\varepsilon +{a}_3{\varepsilon}^2+{a}_4{v}^3+{a}_5{v}^2\varepsilon +{a}_6v{\varepsilon}^2+{a}_7{\varepsilon}^3+O\left({\varepsilon}^4\right) $$

The eigenvalues of the Jacobian matrix, *λ*, in (33) are 0 and 2.9 × 10^−4^. The given center manifold is an invariant manifold that is a tangent space of the center subspace, which is an eigenspace when the eigenvalue is equivalent to zero. The behavior of the fluctuation is complex when the real part of the eigenvalue is equivalent to zero. The above result in (36) shows that it is systematically necessary to analyze the behavior of the given system on the center manifold [22]. In order to analyze the behavior of the system, we investigated whether the change of the value in *p* around the critical value *p*_c_ gives *u* that satisfies *du/dt* = 0. When the two values of *u* are given, i.e., bifurcation of the system is shown, and oscillation and/or other interesting behaviors may be predicted.

Using (40), we obtained:

41 $$ u=\left(dv/dt\right)\partial h\left(u,\;\varepsilon \right)/\partial u+\left(d\varepsilon /dt\right)\partial h\left(u,\;\varepsilon \right)/\partial \varepsilon =\left(2{a}_1\;v+{a}_2\varepsilon \right){f}_u\left(u,\;v\right) $$

Using Eqs. (40) and (41), we then obtained:

42 $$ \left(2{a}_1\;v+{a}_2\varepsilon \right){f}_u\left(u,\;v\right)={a}_1{v}^2+{a}_2v\varepsilon +{a}_3{\varepsilon}^2+{a}_4{v}^3+{a}_5{v}^2\varepsilon +{a}_6v{\varepsilon}^2+{a}_7{\varepsilon}^3+O\left({\varepsilon}^4\right) $$

Solving Eq. (42) gives the coefficients of *ai* (1 ≤ *i* ≤7) in Eq. (40): *a*3 = *a*7 = 0. Substituting *u* in Eq. (40) given by *ν* and *ε* into *fv* (*u,v*), we obtained the *kinetic stability equation* for fluctuation *ν* using the coefficients *n*_*i*_ (*i* = 1 …, 7) as follows_:_

43 $$ dv/dt={n}_1{v}^2+{n}_2\;v\varepsilon +{n}_3{\varepsilon}^2+{n}_4{v}^3+{n}_5{v}^2\varepsilon +{n}_6v{\varepsilon}^2+{n}_7{\varepsilon}^3+O\left({\varepsilon}^4\right) $$

Independent of the numerical values in Eq. (43),

44 $$ {n}_3,{n}_6,{n}_7=0 $$

Then, we obtained_:_

45 $$ dv/dt={n}_1{v}^2+{n}_2\;v\varepsilon +{n}_4{v}^3+{n}_5{v}^2\varepsilon +O\left({\varepsilon}^4\right) $$

By setting left-hand side equivalent to zero,

46 $$ \begin{array}{l}v=0,\\ {}\left(-{n}_1-{n}_5\upvarepsilon \pm {\left({\left({n}_1+{n}_5\upvarepsilon \right)}^2-4{n}_2{n}_4\upvarepsilon \right)}^{1/2}\right)/2{n}_4\end{array} $$

We obtained an approximate solution to Eq. (46):

47 $$ \begin{array}{l}v=0,\\ {}-2{n}_1+\left(2{n}_2{n}_4/{n}_1-2\kern0.28em {n}_5\right)\upvarepsilon, -2{n}_1\kern0.28em {n}_2\kern0.28em \upvarepsilon /{n}_4\end{array} $$

From (40), we obtained the formulation of *u* using a constant coefficient *c’*,

48 $$ u\approx 0,c'{\left({n}_1/{n}_4\right)}^2 $$

When *D*_*1*_ *, k, p* are sufficiently small, substituting [**l**_**1**_ **l**_**2**_] in (35) into (33) approximately gives :

49 $$ x=-\left( aX/k\right)u+v\sim v $$

As a result, as we described *v* and *x* had two amplitudes in (47) demonstrating the oscillation of the fluctuation by bifurcation in *v*-ε plane (Fig. 2). Thus, stability analysis enables prediction of the behavior of the fluctuation around the critical point of the protein assembly system.

## Discussion

In this work, we presented a model for protein assembly kinetics and analyzed the stability around the critical point using CMT. The nonlinear kinetic equations include three parameters (*X*, *Y*, and *Z*); however, only two are independent. In the simulations, ATP/GTP- or ADP/GDP-binding monomers periodically exhibit an oscillation between assembly and disassembly. This accurately reflects the microtubule kinetics showing unstable assembly [8].

To the best of our knowledge, this is one of the first reports on the application of CMT to the analysis of biological reaction systems [8]. The fluctuation of monomer concentrations was subjected to a perturbation expansion using a minimal increase in the supply of ATP/GTP near the concentration at the critical point. This mathematical method precisely treats nonlinear and multi-parameter systems around the critical point. The fluctuation kinetics is expected to change from convergence to divergence of the concentration fluctuation of the monomer, i.e., from stable to unstable around the critical point, as shown in Fig. 2. Because of this high sensitivity to the concentration of ATP/GTP, protein assembly is dynamically regulated by minimal changes in the supply of ATP/GTP, which in turn is subject to metabolic control. Via modeling of microtubule growth at the mesoscopic scale, Zapperi et al. showed the time course of transition between slow growth and rapid shrinkage during microtubule polymerization [16]. The present simulation may explain microscopic tubulin oligomerization oscillations during the initial steps of microtubule assembly. In addition, the present model may explain the transition from microscopic oligomerization and aggregation to mesoscopic scale assembly. The quantitative evaluation of the theoretical basis of protein assembly requires further investigation through experimental studies.

The present center manifold analysis enables elucidation of detailed behavior around the steady state and oscillatory dynamics of protein monomer concentration. In the current study, we further developed the mathematical framework using CMT and aimed to describe Hopf-bifurcation around the steady state, through the center manifold analysis, in a simple model. Coveney et al. have described a detailed model of protein assembly, including nucleation, its catalysis, and inhibition processes and performed a kinetic analysis of the initial nucleation process [23, 24]. The kinetic model of monomer-oligomerization or nucleation requires multiple concentrations that describe variable oligomer and nucleation. As shown by Coveney et al., it was challenging to predict the behavior of the system using a multi-parametric (dimensional) center-manifold on the model. In the current study, we utilized a monomeric parameter and showed bifurcation of the system around the critical point. Therefore, CMT in a simple model serves to reduce the dimensions of the system to signal dimensions, as shown in this study. We expect that the theoretical framework in the current study provides a general theory of protein assembly kinetics and signal transduction [5, 25].

The analysis of growth kinetics of polymerization, according to Oosawa’s model, has recently been reported by Michaels et al. [12]. The authors focused primarily on the dynamic phase of protein polymerization. As nucleation and polymerization to the nucleus proceeds in parallel, the analysis requires a detailed kinetic model of interaction between the nucleation and polymerization process [13, 14]. However, after the dynamic phase and before the plateau phase of polymerization, PM interactions are dominant during signal transduction. The present analysis illustrates the dynamics of cytoplasm in the stable state, and the corresponding influence on cell motility.

The present simulation was applied to such a quasi-statistic state, and the results revealed a possibility that oscillation of monomer concentration may occur when the ATP/GTP concentration exceeds the critical concentration. The calculated critical concentration of ATP/GTP, based on Hopf-bifurcation in (46) and amplitude of the fluctuation, coincided well with the amplitude obtained via the present simulation. The consistency in values in the simulation is important for verification. The periodic change in concentration may contribute to the coherently spatial-periodic viscosity and subsequently to contraction and elongation during cell movement. A recent study demonstrated the role of cytokeratin in determining keratinocyte motility and shape [26] and experimental method has greatly developed [27]. Structural components of cells determine non-linear cellular structural behavior and the contribution of various cell components to stability in response to mechanical stimuli. The cytoskeleton plays key roles in determining cellular stiffness. Our model captures non-linear structural behaviors including variable compliance along the cell surface and resistance to pull-out force [28]. The role of the microtubules in dynamic behavior may be investigated from the viewpoint of cell geometries. Measurement of the oscillation and determination of the critical concentration of ATP/GTP may reveal physical properties such as elasticity and compressibility.

## Conclusion

Our model is expected to be useful for computing biophysical behavior in response to minute changes in GTP/ATP concentration using fluorescence intensity meter in two-dimensional cell geometries. In addition, the present model is expected to be suitable for use in algorithms for simulation of metabolic processes. Although further experimental studies are necessary for verification, our findings show that the current non-linear model of dynamic instability analysis captures the non-linear behaviors of cellular chemical and mechanical responses.

## Abbreviations

ATP: Adenosine triphosphate ATP
CMT: Center manifold theory
GTP: Guanidine triphosphate
PM: Polymer and monomer (PM)
